# A Straight Talk on Hospital Finances

**Published:** 1918-01-05

**Authors:** James W. Lowther

**Affiliations:** Speaker of the House of Commons


### THE FASHION TO DECRY VOLUNTARYISM.

### A Straight Talk on Hospital Finances.

**By the Right Hon. JAMES W. LOWTHER, M.P., Speaker of the House of Commons.**

Lord Farquhar, my lords, ladies, and gentlemen,—You call my remarks "delivering an address," but delivering an address is a rather serious matter. If you had asked me to "say a few words" it would have been more appropriate. You might conclude from what Sir William Collins said that I am in the habit of making speeches in the House of Commons; but Sir William has himself made far more speeches than I have ever made. My misfortune is that I have to remain silent while I hear him speaking. There are many occasions on which I hear speeches when I would very much like to interrupt, and offer some observations in reply. But my position prevents my doing so. I am here by virtue of the fact that I am, for the time being, one of the statutory Governors of King Edward VII.'s Hospital Fund. During the minority of H.R.H. the Prince of Wales, and during the time he remains abroad, his office in regard to this Fund has devolved upon three of us. I happen to be one of the three, and have taken a more or less active part in the work connected with the work of King Edward VII.'s Fund. I desire, in that capacity, to tender to the League of Mercy, both collectively and individually, our most sincere thanks for the generous support which we have again received at your hands this year. The figures have already been mentioned. You have, again this year, been able to give us a contribution of £15,000. More remarkable still, I think, is the fact that since you have been founded you have given us already more than a quarter of a million pounds. That, I think, is one of the most remarkable things in relation to this Fund which can be brought before the notice of the public. Starting, as you did, with a gift of £1,000 in the first year, you have now increased it to £15,000. I remember that a year or so after the war began—it was the first meeting of King Edward VII.'s Fund Council after the war—it fell to my lot to make a speech on that occasion. I then said I thought, considering the enormous claims which the war made upon all charitable persons, the enormous claims in war-time there are from all quarters besides those for existing hospitals, we should have to look for some slight reduction in the amount which would be available for distribution. At the time, Sir Henry Burdett, who is a very active member of the Council of King Edward VII.'s Fund, contradicted me, saying he did not think so at all: he thought the probability was that fully as much money would be collected and be available, possibly even more. I had my doubts at the time, but I am glad now to be able to confess that I was wholly wrong, and that he was right. It is not always pleasant to admit that one is wrong, but on this occasion it is a great pleasure to me to confess that he had formed a much juster conception of the charitable nature of our fellow-citizens, their organisation, their capacity for work, the energy which the League of Mercy can put, and has put, into this work.

A fashion in these days is, I think, somewhat to decry voluntaryism. The demand has been made, and we hear it repeated from time to time, that the hospitals should be put upon the rates. It is pointed out that the hospitals are available for all persons throughout the Metropolis, and that therefore all the ratepayers should be called upon to support them, and that it is not fair that the hospitals, which are bound to take in the sick poor throughout London, should be supported entirely by a small minority, the benevolent and charitable persons. I confess that, personally, I am not in favour of any such change. I think that as long as we find them well-conducted, as long as we find devoted medical people giving their time, their attention, and their abilities to the strenuous work which the hospitals entail, as long as we find the charitable ready to support with their alms these absolutely essential institutions, and so long as some such authority as King Edward VII.'s Fund is prepared to see that these funds are economically, frugally, and properly administered, I think it would be a great mistake to put upon the rates the support of these institutions. (Hear, hear.) It is, no doubt, easier to take out your cheque-book and draw a cheque than it is to do the work which the members of your League are doing. We have heard to-day of the great exertions which they have undertaken in respect to the collection of funds by various methods: house-to-house collections, bazaars, kinemas, and so forth. That is all splendid and useful work, and I think it would be very undesirable indeed to put that work upon the ratepaying public. It would be a great mistake, because you have got here the energy, you have got organising talent, you have got the will to do the thing; and it would be a pity if all those qualities were to be wasted and go for nothing, and that persons should be paid to do the work which many are ready and willing to undertake voluntarily. And I am not sure, either, that the administration of rates which are easily raised is always very economical. The popular idea, I suppose, is that the representatives of the ratepayers give a very close attention to the method in which the rates are expended. It is a sound theory, I dare say, but it does not always hold. And I think that in considering the administration of our hospitals and the method in which that has, during the last few years, been super-

**A STRAIGHT TALK ON HOSPITAL FINANCES.**—(*continued*).

vised, we have a complete safeguard that the money which is given is economically and well administered, and I should be very sorry to see any change. It is a remarkable thing that the other great voluntary organisation which still exists in the State is the Lifeboat Institution, and this, like the hospitals, is engaged in the work of saving life; they still remain the chief features of voluntaryism in this country.

You, the League of Mercy, are what I may really call the working partner of King Edward VII.'s Fund. We have heard from your hon. treasurer of the splendid spade-work of the League, which no doubt will continue to be done; the house-to-house canvass, and so forth. It is rather bold of me, perhaps, to make the suggestion—it may be it is already being carried out—but there are now large collections of people, in munition works and offices in London, who, to say the least, are getting very fair remuneration for their work, and who, if we can believe all we see in the papers, seem to spend their money very freely. I do not know what the authorities would say to it, but would it not be possible to institute collections in those establishments? I know that in some offices collections are permitted for all sorts of objects which are not nearly so deserving—for wedding gifts for those who are so fortunate as to get married, testimonials to the superintendents who are leaving, or who are being promoted to more lucrative offices, and so on. If those are permitted, I should think there would be no hesitation as to granting leave to collect, under certain well-defined conditions and restrictions, funds for such a worthy object as the League of Mercy. Your duty, as has been pointed out, is to collect in your net little fish.

We collect the big fish in King Edward VII.'s Fund. When we get a gift of £20,000 or £30,000 there, we say "Thank you" as pleasantly as we can, but we are not surprised at it. I do not say we get such a sum every day, but we are constantly getting very large gifts. It is through you only we can get into touch with the vast majority of the inhabitants of the Metropolis. Through His Majesty himself and through some of the very distinguished members of the Council we get into touch with the very rich people, and their gifts come to us direct. But you are the working machinery who are able to grind for us those smaller coins which are absolutely necessary for carrying on the work which we have in hand. Every time I see the accounts of this marvellous generosity I wonder more and more "where the money comes from." We never knew, until the war had been going on for some time, what a very rich people we were; we never knew until then, I think, what a very generous people we were. When we look at the Prince of Wales's Fund and all the other funds which have been compiled and see them increasing in size, we stand perfectly aghast at the great wealth and the great munificence of this country. I thought it was quite possible that, the attention of the public being so much directed to the war, and the desire of everybody being to help the soldiers as much as they possibly could, the wants of the civilian population might have been forgotten; and it is a great satisfaction to us on King Edward's Fund—and I am sure it is to you also—to find that that is not so, and that, notwithstanding all the sums of money which are collected and given to help our brave soldiers who come back wounded and disabled, that the wants of the civil population still find a warm place in the hearts of their fellow-citizens. The title of the League of Mercy reminds me that Shakespeare said that "mercy is twice blest." I think he did not mean it in the sense in which you employ the term "mercy" in connection with the title of your League; when he said "It blesseth him that gives and him that takes" he meant it blesses the people who subscribe, and it is also a blessing to those who are the means by which those subscriptions are collected. I believe it produces a great sense of satisfaction and of pleasure to everybody to be able to feel that they are doing something which is useful to their fellow-countrymen by the work and the energy which they put into the collections which they make on behalf of the League of Mercy.

I conclude, as I began, by thanking you most sincerely on behalf of the King's Fund for the most splendid work you have done in the past, and I wish you all success in the future. (Loud applause.)

